# Dynamic compression schemes for graph coloring

**DOI:** 10.1093/bioinformatics/bty632

**Published:** 2018-07-18

**Authors:** Harun Mustafa, Ingo Schilken, Mikhail Karasikov, Carsten Eickhoff, Gunnar Rätsch, André Kahles

**Affiliations:** 1 Department of Computer Science, ETH Zurich, Zurich, Switzerland; 2 Biomedical Informatics Research, University Hospital Zurich, Zurich, Switzerland; 3 SIB Swiss Institute of Bioinformatics, 1015 Lausanne, Switzerland; 4 Brown Center for Biomedical Informatics, Brown University, Providence, RI, USA

## Abstract

**Motivation:**

Technological advancements in high-throughput DNA sequencing have led to an exponential growth of sequencing data being produced and stored as a byproduct of biomedical research. Despite its public availability, a majority of this data remains hard to query for the research community due to a lack of efficient data representation and indexing solutions. One of the available techniques to represent read data is a condensed form as an assembly graph. Such a representation contains all sequence information but does not store contextual information and metadata.

**Results:**

We present two new approaches for a compressed representation of a graph coloring: a lossless compression scheme based on a novel application of wavelet tries as well as a highly accurate lossy compression based on a set of Bloom filters. Both strategies retain a coloring even when adding to the underlying graph topology. We present construction and merge procedures for both methods and evaluate their performance on a wide range of different datasets. By dropping the requirement of a fully lossless compression and using the topological information of the underlying graph, we can reduce memory requirements by up to three orders of magnitude. Representing individual colors as independently stored modules, our approaches can be efficiently parallelized and provide strategies for dynamic use. These properties allow for an easy upscaling to the problem sizes common to the biomedical domain.

**Availability and implementation:**

We provide prototype implementations in C++, summaries of our experiments as well as links to all datasets publicly at https://github.com/ratschlab/graph_annotation.

**Supplementary information:**

Supplementary data are available at *Bioinformatics* online.

## 1 Introduction

The revolution of high-throughput DNA sequencing has created an unprecedented need for efficient representations of large amounts of biological sequences. In the next five years alone, the global sequencing capacity is estimated to exceed one exabyte (Stephens *et al*., 2015 ). While a large fraction of this capacity will be used for clinical and human genome sequencing, such as the 1000 Genomes Project (Auton *et al*., 2015) or the UK10K (Walter *et al*., 2015) effort, that are well suited for reference-based compression methods, the remaining amount is still dauntingly large. This remainder does not only include sequences of model and non-model organisms (Zhang *et al*., 2015) but also community approaches such as whole metagenome sequencing (WMS) (Ehrlich and Consortium, 2011; Turnbaugh *et al*., 2007).

The next logical steps of data integration for genome sequencing projects are assembly graphs that help to gather short sequence reads into genomic contigs and eventually draft genomes. While assembly of a single species genome is already a challenging task (Bradnam *et al*., 2013), assembling a set of genomes from one or many WMS samples is even more difficult, with preprocessing methods such as taxonomic binning (Dröge and McHardy, 2012) helping to reduce its complexity. A commonly used strategy to generate sequence assemblies is based on de Bruijn graphs that collapse redundant sequence information into a set of unique *k-*mers, substrings of length *k* (Pevzner *et al*., 2001). Especially in a co-assembly setting, where sequences and meta-information from multiple source sequence sets is combined and stored, *colored* de Bruijn graphs form a suitable data structure, as they allow association of multiple colors with each node or edge (Iqbal *et al*., 2012). In this paper, we use a definition of this graph specific to the field of bioinformatics. More precisely, a *graph coloring* is a graph edge labeling that assigns an arbitrary number of distinct colors to each edge of the graph. The set of colors assigned to an edge is called the *edge coloring*. Note that we imply no additional restrictions on the graph coloring (i.e., neighboring edges are allowed to have same colorings). Another important application of colored de Bruijn graphs is building an efficient representation and indexing of multiple genomes, forming a so-called *pan-genome* store (Myers *et al*., 2017).

Owing to the large size, and, subsequently, the excessive memory footprints of such graphs, recent work has suggested compressed representations for de Bruijn graphs based on approximate membership query (AMQ) data structures (Benoit *et al*., 2015; Chikhi and Rizk, 2013) or generalizations of the Burrows-Wheeler transform to graphs (Bowe *et al*., 2012). The recent work on compressed colored de Bruijn graphs has followed this trend. Currently, there exist two distinct paradigms. The first is to compress the colored graph in a single data structure while the second proposes two separate (compressed) representations of a graph and its coloring. The first group contains approaches such as *Bloom Filter Tries* (Holley *et al*., 2016) for pan-genome representation, *deBGR* (Pandey *et al*., 2017a) that encodes a weighted de Bruijn graph, or Split Sequence Bloom Trees (Solomon and Kingsford, 2017) that index short read datasets based on a hierarchically structured set of Bloom filters.

Approaches that fall into the second group usually encode graph coloring as a compressed binary matrix (an *annotation matrix*), and include *VARI* (Muggli *et al*., 2017), which uses succinct Raman-Raman-Rao or Elias-Fano compression on the annotation matrix, and *Rainbowfish* (Almodaresi *et al*., 2017), which additionally takes into account the distribution of the unique edge annotations in the graph to achieve better compression ratios. A very recent addition that shows features of both groups is *Mantis* (Pandey *et al*., 2017b), which re-purposes the integer counts in a counting AMQ data structure to act as keys in a color-class table.

Our contribution falls into the second group and allows for efficient addition and removal as well as editing of individual annotation tracks (individual colors) on an existing graph structure. We present a data structure for annotation matrix compression based on *wavelet tries* that takes advantage of correlations between matrix columns and achieves excellent compression ratios on a wide range of input data. Moreover, the proposed data structures can efficiently handle dynamic settings where coloring or underlying graph structure are subject to change.

For genomics applications, where an exact reconstruction of the coloring is not necessary but an approximate recovery with high accuracy would be sufficient, we also present a probabilistic compression scheme for an arbitrary number of colors. Possible use cases for such a scenario are the taxonomic classification of sequencing reads or the identification of approximate matches in a large database of sequences, e.g., the lookup of a sequence marker. As the graph stores the exact sequence information, approximate labeling is often sufficient. Based on Bloom filters (Bloom, 1970), a data structure for efficient AMQ with a one-sided error, we encode colors as bit vectors and store them in a set of filters. We further reduce the necessary storage requirements of the individual filters by maintaining weak requirements on their respective false-positive rates, which is subsequently corrected for using neighborhood information in the graph.

Although both proposed techniques for color compression take advantage of the underlying sequence graph, they impose no restrictions on its topology.

## 2 Approach

We consider a *colored de Bruijn graph* (cDBG) that represents a set of biological sequences and their metadata. It consists of a node-centric de Bruijn graph (in which each node is an observed *k*-mer) constructed from the collection of input sequences (forward and reverse complement) and an *annotation* associated with the *k*-mers generated from these input sequences. The annotation can consist of several colors, each representing a label to a *k*-mer, e.g., whether it is found in a certain species. We represent this annotation as a binary matrix, where each row corresponds to an edge and each column corresponds to a color. Set bits in this matrix indicate associations of edges with colors.

### 2.1 Preliminaries and notation

Let Σ be an alphabet of fixed size (in the case of genome graphs, Σ={A,C,G,T,N}). Given a string $s\in \Sigma^{*}$, we use s[i:j] to denote the substring of *s* from index *i* up to and including index *j*, with i,j≥1.

Given a bit vector $b\in {\left\{0,1\right\}}^{m}$ of length *m*, we use the notation |b| to refer to its length, b[i] to refer to its $i^{\text{th}}$ character, 1≤i≤|b|, b[j:k] to refer to the bit vector b[j]⋯b[k], b[:k] to refer to its prefix b[1:k], and b[j:] to refer to its suffix b[j]⋯b[|b|]. The empty vector is denoted *ε*. Finally, given bit vectors $a,b\in {\left\{0,1\right\}}^{m}$, we use the notation a∨b and a∧b to denote the bitwise OR and AND operators, respectively.

The function ${\text{rank}}_{0}(b,j)$ counts the occurrences of the character 0 in the prefix b[:j], while ${\text{select}}_{0}(b,j)$ returns the index of the $j^{\text{th}}$0 in b. The functions ${\text{rank}}_{1}{\;\text{and}\;\text{select}}_{1}$ are defined analogously for the 1 character. We will use the notation $2^{A}$ to denote the power set of a set *A* and abuse the notation |·| to also denote set cardinalities.

### 2.2 Graph representation

Given an ordering of the edges $E=(e_{1},\ldots ,e_{n})$ of an underlying graph G=(V,E) and a set of colors 1,…,m, we define the *annotation matrix*$A\in {\left\{0,1\right\}}^{n\times m}$ such that

(1) $$A_{j}^{i}=1_{\left\{e_{i}\;\;\text{has}\;\text{color}\;\;j\right\}}=\{\begin{array}{cc} 1, & e_{i}\;\;\text{has}\;\text{color}\;\;j,\\ 0, & \;\text{otherwise}. \end{array}$$

As a proof of concept for the graph coloring presented in this work, we use a simple representation of a de Bruijn graph with its edges (the *k*-mers) stored in a hash table.

During construction of the graph, the edge colorings are computed based on the metadata of the input sequences. We assign to each unique metadata string a positive integer index (*color*). During *k*-mer enumeration, each *k*-mer is assigned to a set of colors encoding its respective metadata strings. We then represent this coloring through a binary vector (*bit vector*) with bits set for the corresponding edge colors. When duplicate *k*-mers are collected to construct graph edges, we combine the *k*-mers’ respective bit vectors via bit-wise OR operations and assign the aggregated coloring to the resulting edge. Alongside the de Bruijn graph, this process results in the encoding of a *graph coloring* as an annotation matrix A with n rows corresponding to the edges of the graph and *m* columns corresponding to the total number of colors observed during construction. The resulting graph-annotation pair (G,A) is a *colored de Bruijn graph*. When the graph is queried, search patterns are mapped to a path (a sequence of edges) and, hence, to a corresponding sequence of annotation matrix rows.

### 2.3 Graph coloring compression

#### 2.3.1 Lossless row compression with wavelet tries

**Fig. 1.**
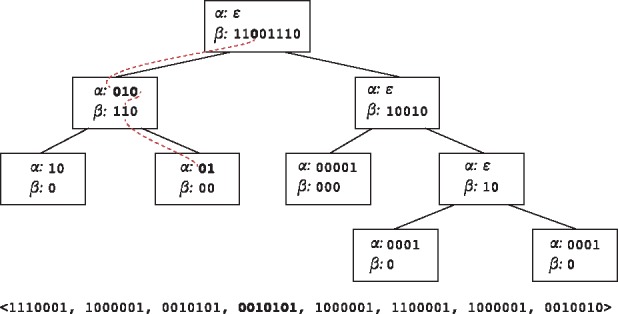
A wavelet trie constructed for a tuple of bit vectors. Each node is labelled with a *longest common prefix* (LCP) *α* and an *assignment vector β*. During construction at a particular node, the LCP of the bit vectors is extracted and the next significant bit is used to assign the bit vector suffixes to that node’s children. A node becomes a leaf when all bit vectors assigned to it are equal. An example is given in bold. The sequence 0010101 results from the traversal along the dashed line from top to bottom. The index *i* being queried is updated by calling rank_0(·,i)_ (*i*) (traverse left) or rank_1(·,i)_ (*i*) (traverse right) on the *β* vectors

For lossless compression of annotation matrices, we propose a novel application of the *wavelet trie* data structure (Grossi and Ottaviano, 2012). Wavelet tries compress tuples of dynamic bit vectors by finding their shared contiguous subvectors (Fig. 1). Briefly, a wavelet trie builds on the concept of a wavelet tree and takes the shape of a compact prefix tree (a binary radix trie). A set of bit vectors (in our case representing the annotations) is encoded as paths from the root to the leaves of the tree, storing prefixes shared by all children of a node only once. Querying a bit vector at index *i* is done by tree traversal starting at the root by concatenating the shared subvectors stored at each node.

In the context of genome graph coloring, we employ wavelet tries to compress the rows of the annotation matrix to allow for dynamic updates in its rows and columns. We employ a construction strategy based on wavelet trie merging (Böttcher *et al*., 2017; Grossi and Ottaviano, 2012), but in a parallel fashion. Their merging algorithm assumes that the set of bit vectors being compressed is *prefix free* (i.e., that no vector is a prefix of another vector), which in our case, is not necessarily true. For our method, we maintain the property that all bit vectors are of the same length by right-padding with 0s.

**Construction**: The wavelet trie encoding the annotation matrix $A\in {\left\{0,1\right\}}^{n\times m}$ is constructed recursively and is a binary tree (Fig. 1) with nodes *V_T_* of the form

$$(\alpha_{j},\beta_{j})\in V_{T},\;\alpha_{j},\beta_{j}\in {\left\{0,1\right\}}^{*}.$$

The *α_j_* are referred to as the *longest common prefixes* (LCPs) and the *β_j_* are referred to as the *assignment vectors*.

We define the initial tuple of input bit vectors to be the rows of A, $B=(A^{1},\ldots ,A^{n}),\;\text{where}\;A^{i}=(A_{1}^{i},\ldots ,A_{m}^{i})\in {\left\{0,1\right\}}^{m},\;1\leq i\leq n.$ The algorithm starts by constructing the *root* node $\left(\alpha_{1},\beta_{1}\right)$ from the initial set of input vectors $B_{1}:=B$.

Beginning with $j=1\;\text{and}\;\ell_{j}=|B_{j}|$, for a list of input bit vectors

$$B_{j}=(b_{j}^{1},\ldots ,b_{j}^{\ell_{j}}),\;\;b_{j}^{i}\in {\left\{0,1\right\}}^{k_{j}},\;\;1\leq i\leq \ell_{j},$$

we compute $\left(\alpha_{j},\beta_{j}\right)$ as follows. First, we compute the longest common prefix $\alpha_{j}:=LCP(B_{j})$ for the bit vectors in *B_j_*, defined as,

$$LCP(B_{j})=\underset{{}_{\left\{\alpha \in {\left\{\text{0},\text{1}\right\}}^{\text{*}}\;|\;b_{j}^{i}[:\text{|}\alpha \text{|}]\text{=}\alpha \;\forall i\mathrm{=1},\ldots ,\ell_{\text{j}}\right\}}}{\text{arg max}}\text{|}\alpha \text{|}.$$

If the computed *α_j_* is identical to all the input bit vectors, let the assignment vector consist of $\left|B_{j}\right|$ zeros, $\beta_{j}:=(0,\ldots ,0)$ and terminate the recursion branch. $\left(\alpha_{j},\beta_{j}\right)$ is referred to as a *leaf.* Otherwise, the assignment vector is set to be the concatenation of the next significant bits in each of the $b_{j}^{i},\;1\leq i\leq \ell_{j}$ after removing the common prefix *α_j_*,

$$\beta_{j}:=(b_{j}^{1}[|\alpha_{j}|+1],\ldots ,b_{j}^{\ell_{j}}[|\alpha_{j}|+1]).$$

We continue the recursion on the child nodes $\left(\alpha_{2j},\beta_{2j})\;\text{and}\;(\alpha_{2j+1},\beta_{2j+1}\right)$, with the new tuples of bit vectors $B_{2j}\;\text{and}\;B_{2j+1}$, respectively, which are defined by partitioning *B_j_* based on the assignments *β_j_* and removing the first $|\alpha_{j}|+1$ bits,

$$\begin{array}{c} B_{2j}:=(b_{j}^{{\text{select}}_{0}(\beta_{j},1)}[|\alpha_{j}|+2:],\ldots ,\\ b_{j}^{{\text{select}}_{0}(\beta_{j},{\text{rank}}_{0}(\beta_{j},|\beta_{j}|))}[|\alpha_{j}|+2:]);\\ B_{2j+1}:=(b_{j}^{{\text{select}}_{1}(\beta_{j},1)}[|\alpha_{j}|+2:],\ldots ,\\ b_{j}^{{\text{select}}_{1}(\beta_{j},{\text{rank}}_{1}(\beta_{j},|\beta_{j}|))}[|\alpha_{j}|+2:]). \end{array}$$

Parallel construction via trie merging: To allow for parallel construction from batches of edge colorings, we develop a variant of the algorithm to merge wavelet tries presented by Grossi and Ottaviano (2012) and Böttcher *et al*. (2017). Merging proceeds by performing an *align* and a *merge* step on each node, starting from the root (Supplementary Section A and Fig. S1). Given two wavelet tries $T\prime \;\text{and}\;T^{\prime \prime }$ with node sets $V_{T\prime }={\left\{(\alpha \prime_{j\prime },\beta \prime_{j\prime })\right\}}_{j\prime =1}^{n\prime }$ and $V_{T^{\prime \prime }}={\left\{(\alpha_{j^{\prime \prime }}^{\prime \prime },\beta_{j^{\prime \prime }}^{\prime \prime })\right\}}_{j^{\prime \prime }=1}^{n^{\prime \prime }}$ that we want to merge into a new trie *T*, the merging process can be summarized in three steps:

1. **Align:** For the nodes $\left(\alpha \prime_{j\prime },\beta \prime_{j\prime }\right)$ and $\left(\alpha_{j^{\prime \prime }}^{\prime \prime },\beta_{j^{\prime \prime }}^{\prime \prime }\right)$, compute the longest common prefix $\hat{\alpha }=LCP(\alpha \prime_{j\prime },\alpha_{j^{\prime \prime }}^{\prime \prime }).$ For each of $V_{T\prime }\;\text{and}\;V_{T^{\prime \prime }}$, replace their respective children with a new child inheriting these children, set the *α* values of the new children to the non-LCP parts of $\alpha \prime_{j\prime }\;\text{and}\;\alpha_{j^{\prime \prime }}^{\prime \prime }$ and the *β* values to $\beta \prime_{j\prime }$ and $\beta_{j^{\prime \prime }}^{\prime \prime }$, respectively. Replace $\alpha \prime_{j\prime }\;\text{and}\;\alpha_{j^{\prime \prime }}^{\prime \prime }\;\text{with}\;\hat{\alpha }$ and choose appropriate *β* vectors.
2. **Merge:** As $\alpha \prime_{j\prime }\;\text{and}\;\alpha_{j^{\prime \prime }}^{\prime \prime }$ are now equal, concatenate $\beta \prime_{j\prime }\;\text{and}\;\beta_{j^{\prime \prime }}^{\prime \prime }$.
3. **Repeat:** Move to the children of j′ and $j^{\prime \prime }$ and apply the same function until all leaves are reached.

In general, this algorithm can be used to insert rows in arbitrary positions of the annotation matrix by inserting into appropriate positions in *β*. This can also be used to update entries in a compressed matrix by removing that row and internally inserting a modified row (see Supplementary Section C.1.4).

**Time complexity:** Let $A\in {\left\{0,1\right\}}^{n\times m}$ be the annotation matrix. The height of a constructed wavelet trie with nodes *V_T_* depends on the degree to which the input bit vectors share common prefixes. Since there can be at most *n* leaves, and the maximum height of the trie is at most *m*, the number of nodes can be at most $\left|V_{T}|\leq \min(2n-1,2^{m}-1\right)$.

Given two wavelet tries with sets of nodes $V_{T^{\prime }}\;\text{and}\;V_{T^{\prime \prime }}$, merging is performed in $O(|V_{T\prime }|+|V_{T^{\prime \prime }}|+|\beta_{1}^{\prime }|+\beta_{1}^{\prime \prime }|)$ time (Grossi and Ottaviano, 2012). Once a wavelet trie is constructed, queries can be performed in O(h) time, where h≤m is the height of the trie. To achieve this value, the *β_j_* are compressed with RRR coding (Raman *et al*., 2007) to support rank operations in O(1) time.

**Using prior knowledge to improve compression**: One of the most important factors determining the compression ratio (see Section 2.4 for a formal definition) of a wavelet trie is the distribution of longest common prefixes encountered during construction. We explore whether prior knowledge can be used to form groups of similarly colored edges and help optimize compression ratios.

Given a similarity metric defined on edge colorings, edges can be grouped into *classes* defined by high similarity between their constituent edge colorings. Example class definitions can be based on phylogenetic information (e.g., shared taxonomic IDs) or sequence alignment information (e.g., alignment to a given reference genome).

To encode the assignment of edges to classes, we introduce additional colors called *class indicator bits* and add corresponding new columns to the annotation matrix. Additionally, we hypothesize that if the indicator columns are of low index, then edges from the same class are more likely to be co-assigned to matching nodes in a wavelet trie. This would facilitate a partitioning of the rows that has the potential to significantly improve the compression ratio of the wavelet trie by facilitating grouping of similar rows closer to the tree root. We implement this procedure by providing class information as additional metadata strings, which are then used to augment the coloring of each edge with the color of its corresponding class.

Such class information can be either an encoding of prior knowledge, such as phylogenetic distance or sample similarity, or if such information is unavailable a measure for the expected similarity between the sequences of any two given colors, which could be estimated using sketching techniques such as minimal hashing (Ondov *et al*., 2016).

#### 2.3.2 Probabilistic column compression with Bloom filters

For cases where a lossy compression scheme with moderate loss of accuracy will suffice in place of fully lossless compression, we explore a probabilistic compression of the annotation matrix as a near-exact compromise. Since, by definition, the columns of the annotation matrix encode set membership, it is possible to compress them using Bloom filters (Bloom, 1970), a probabilistic data structure for approximate set membership queries.

A *Bloom filter* is a tuple BF=(B,H), where $B\in {\left\{0,1\right\}}^{b}$ is a bit vector and $H=\{h_{1},\ldots ,h_{d}\}$ is a collection of *d* hash functions mapping each input to an element of {1,…,b}. For simplicity of notation, let $e_{i}\in {\left\{0,1\right\}}^{b}$ denote a bit vector in which only the $i^{\text{th}}$ bit is set to one.

**Construction:** Two of the operations supported on this structure are insert and the relation of approximate membership ∈,

$$\begin{array}{c} \text{insert}((B,H),x)=\left(B\vee e_{h_{1}(x)}\vee \cdots \vee e_{h_{d}(x)},H\right),\\ x\in BF\Leftrightarrow \text{insert}(BF,x)=BF, \end{array}$$

where insert is used to successively hash new elements into the filter.

**Bloom filter reparametrization:** Although the Bloom filter has no false negative errors, the *false positive probability* (FPP) of the approximate membership query on a Bloom filter with *s* inserted elements can be approximated (Mitzenmacher, 2001) as

(2) $$\text{FPP}(b,d,s)={\left(1-{\left(1-\frac{1}{b}\right)}^{ds}\right)}^{d}\approx {\left(1-e^{-\frac{ds}{b}}\right)}^{d}.$$

As a corollary, an alternate parametrization of Bloom filters can be derived. Given a target false positive probability *p* and *s* elements to insert, optimal values for d and b (Mitzenmacher, 2001) are

(3) $$d=\left\lceil -{\;\text{log}\;}_{2}p\right\rceil ,\;b=-s\frac{{\;\text{log}\;}_{2}p}{\ln\;2}.$$

Given an encoding of an annotation matrix $A\in {\left\{0,1\right\}}^{n\times m}$ as a collection of Bloom filters $BF_{1},\ldots ,BF_{m}$, the *raw annotation* of an edge $e_{i}\in E$ being queried is as follows:

(4) $$\text{query}(e_{i})=(1_{\left\{e_{i}\in BF_{1}\right\}},\ldots ,1_{\left\{e_{i}\in BF_{m}\right\}}).$$

**Neighborhood-based Bloom filter correction:** Following the same rationale as for the wavelet tries, and building on the fact that edges neighboring in the graph often share a large proportion of their colors, we introduce an assumption that all nodes in a *linear path* (a directed path in which all inner nodes, i.e., except for the first and last nodes, have exactly one incoming and one outgoing edges) share an identical coloring. For annotations representing membership of input sequences to the source datasets, this assumption can be always satisfied by prepending and appending all the input sequences with a sentinel character, e.g., $. This would implicitly create branchings in linear paths of the de Bruijn graph, within which edge colorings in general could be different. Working under this assumption can also drastically improve the compression power of the Bloom filters. More precisely, given a linear path, we compute the intersection of the colorings of ℓ edges in some neighborhood within the path and obtain a coloring with drastically reduced FPP. We let N(e)⊂E denote the topological neighborhood of cardinality ℓ around an edge e∈E within a linear path in which all nodes are assumed to share the same colorings, and define the *corrected annotation* as

(5) $$\text{annotation}(e)=\text{query}(e)\wedge \underset{e\prime \in N(e)}{\wedge }\text{query}(e\prime ).$$

Following the argument in (Mitzenmacher, 2001) (see Formula 2), the FPP for one color of a segment of length ℓ can be approximated as

(6) $$\text{FPP}{\left(b,d,s\right)}^{\ell }\approx {\left(1-e^{-\frac{ds}{b}}\right)}^{d\ell },$$

since ℓ false positive errors have to be made to lead the overall Bloom filter to a false positive error.

We implement Bloom filter annotation correction as propagation of precomputed edge colorings to their respective neighboring edges. The propagation terminates when the coloring stops changing or the ends of a linear path are reached.

This correction method relies on direct access to the underlying graph structure to reference during decoding, in contrast to the wavelet trie approach in which this is not strictly required.

### 2.4 Data

The datasets used to evaluate the performance of our compression schemes originate either from viruses (*Virus100*–*Virus50000*), bacteria (*Lactobacillus*) or human (*chr22+gnomAD* and *hg19+gnomAD*) and are chosen to test the methods on different color distributions, annotation matrix sizes and densities. They further reflect varying graph topologies and allow us to study the effect of topology-informed compression in a robust testing bed. We construct de Bruijn graphs of order *k *=* *63 for each dataset and compare the compression performance of all methods by measuring the *compression ratio* and time for each dataset, defined as the ratio of the number of bits in an annotation matrix and the number of bits in its respective compressed representation.

Table 1 summarizes all used datasets in terms of their number of nodes and edges for the constructed de Bruijn graphs, as well as their respective numbers of colors and unique edge colorings. Please refer to Supplementary Section C for a more detailed description of the datasets.

**Table 1:** Datasets used for evaluation

Data set	Nodes	Edges (*n*)	Colors (*m*)	Colorings	Density (%) ($\frac{s}{nm}$)
Virus100	2,954,719	2,956,113	100	463	1.056
*Virus1000*	30,310,634	30,347,373	1,000	11,612	0.117
*Virus50000*	622,587,315	625,110,390	53,412	1,359,843	0.006
*Lactobacillus*	134,951,429	135,369,397	135	6,630	1.475
*chr22+gnomAD*	178,196,890	180,023,641	9	510	15.270
*hg19+gnomAD*	5,714,136,751	5,728,489,633	30	380,051	1.762

Columns represent number of nodes and edges per dataset, total number of colors and number of unique edge colorings, or unique rows of the annotation matrix, and density of the annotation matrices, where the quantity *s* refers to the number of set bits in the annotation matrices.

## 3 Evaluation and applications

In this section, we explore our hypothesis that graph topology can aid in improving compression ratios and study the space complexities of our compression techniques on a variety of viral datasets increasing in size. Finally, we compare the compression ratios of our methods to those of general compression algorithms and those of methods developed specifically for de Bruijn graph coloring compression.

Experiments were performed on a single thread for Bloom filter compression and ten threads for wavelet trie compression, on the Intel(R) Xeon(R) CPU E5-2697 v4 (2.30 GHz) cores of ETH’s shared high-performance compute systems. Run times and peak RAM consumption are reported in Supplementary Figure S6.

### 3.1 Graph topology affects compression ratios

For both the wavelet trie and Bloom filter compression schemes, we explored methods for encoding graph topology with the goal of improving compression ratios. To this end, we explore the introduction of class indicator bits for wavelet tries and graph neighborhood-based annotation correction for Bloom filters.

#### 3.1.1 Improving wavelet trie compression using indicator columns

We test the hypothesis that optimal compression can be achieved by setting class indicator bits in low-index positions in annotation matrices (*H*_0_: column ordering does not influence compression ratios when class indicator bits are set) via an exact test by permuting the annotation matrix column order on the *Virus100* and *Lactobacillus* datasets. More precisely, we generate 100 samples by randomly permuting the columns in the annotation matrix and compress the resulting data to approximate the null distribution of compression file sizes across permutations of the matrix column order (Supplementary Fig. S2).

First, when we test the hypothesis without setting class indicator bits, the compressed file size corresponding to the column ordering induced by the graph construction algorithm is found to not be optimal (Supplementary Fig. S2). However, when class indicator bits are set as the matrix column prefixes, the original ordering of columns is optimal with respect to its approximated null distribution, resulting in an empirical *p*-value of *p *<* *0.01.

#### 3.1.2 Improving Bloom filter FPP using neighborhood correction

**Fig. 2.**
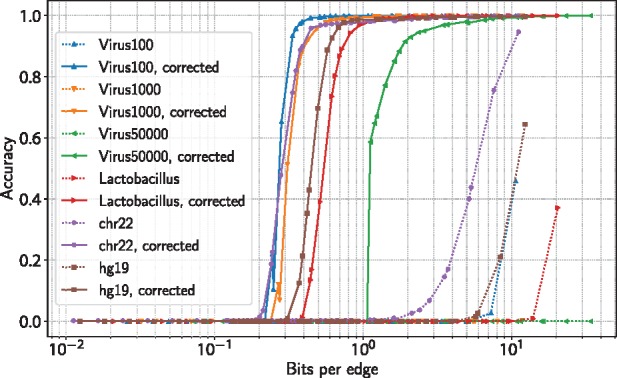
Improvement in Bloom filter compression ratios after neighborhood correction. Bloom filter accuracy (average fraction of correct edge colorings) as a function of filter size (Color version of this figure is available at *Bioinformatics* online.)

We study the effects of neighborhood-based Bloom filter correction on all datasets by varying the average number of bits per edge of the Bloom filters and measuring the accuracy of edge coloring reconstruction (see Section 2.3.2). The results show 70-fold decreases in the number of bits required per edge to achieve similar decompression accuracies on almost all datasets (Fig. 2). A notable exception is the *chr22* dataset, where only a 30-fold improvement is observed.

The average number of linear traversal steps (see Section 2.3.2) needed to correct Bloom filters with sizes ranging from 0.36 to 2.58 bits per edge (Table 2) to an accuracy of 95% ranges from 99.1 to 207.3 (Supplementary Table S1). To correct Bloom filters with sizes ranging from 0.44 to 7.41 bits per edge to an accuracy of 99%, the average number of traversal steps required ranges from 82.3 to 156.3.

**Table 2:** Compression ratio of wavelet trie and Bloom filter schemes (measured as number of bits per edge)

						Proposed			
Data set	Colors (*m*)	gzip	bzip2	VARI	RBF	WTr	WTr (CI)	BF 95%	BF 99.0%
*Virus100*	100	11.4	4.8	9.8	5.8	2.2	1.3 (52)	0.36	0.44
*Virus1000*	1000	26.5	7.5	14.7	9.7	18.2	5.28 (272)	0.49	0.82
*Virus50000*	53,412	135.3	37.7	56.0^a^	^a^^,b^	662.1	64.8 (1693)	2.58	7.41
*Lactobacillus*	135	15.6	5.7	19.3	7.8	3.3	1.6 (20)	0.95	1.40
*chr22+gnomAD*	9	4.6	2.7	17.3^a^	3.3^a^	N/A	1.2 (1)^c^	0.45	2.41
*hg19+gnomAD*	30	10.9	5.4	14.5^a^	5.6^a^	N/A	5.4 (22)^c^	0.68	1.82

*Note:* Each dataset is encoded with eight different compression schemes, including general compression with *gzip* and *bzip2*, existing methods specific to colored de Bruijn graphs *VARI* (Muggli *et al*., 2017) and *Rainbowfish *(RBF, Almodaresi *et al*., (2017)), as well as the wavelet trie encoding (*WTr*) with and without the class indicator bits set (CI; value in parenthesis describes the number of the first columns in the annotation matrices that were used as the indicator columns), and the corrected Bloom filters at >95% (BF *95%*) and >99% (BF *99%*) accuracy. All compression ratios are measured as average number of bits per edge. VARI was compiled with 1024 bit support.

a On these datasets, VARI and RBF results are generated by exporting the annotation data in compatible formats.

b Consumed more than 400GB memory limit.

c The class indicators were the columns representing the reference chromosomes, hence, no extra columns were added.

### 3.2 Properties of compression methods

#### 3.2.1 Compression power grows with the number of colors

**Fig. 3.**
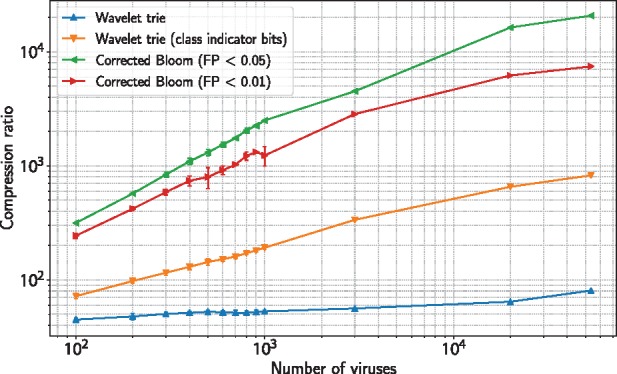
Growth of compression ratios. Compression ratios on virus graphs of increasing genome count. Error bars were computed from the virus graph chains resulting from six random draws of the *Virus1000* dataset (see Section 3.2.1)

To test the scalability of the compression methods, we generate a *chain* (a linear hierarchy) of virus graphs ranging from 100 to 1000 randomly selected genomes in steps of 100 (i.e., $G_{1}\subset \cdots \subset G_{10}$), with additional sets of size 3000 and 20,000, and measure the compression ratios of the annotations for each graph. On our datasets, the wavelet trie method with the addition of class indicator bits and the Bloom filter method with FPP < 0.05 display linear growth in the compression ratio as the number of genomes increases to 1000 genomes (Supplementary Fig. S3), with sublinear growth for more genomes (Fig. 3). Sublinear growth is observed in the wavelet trie method without class indicator bits and, to a lesser extent, the Bloom filter method with FPP < 0.01 (Fig. 3 and Supplementary Fig. S3). A two-fold decrease in compression ratio is observed when the false positive probability criterion for the Bloom filters is decreased from 0.05 to 0.01.

#### 3.2.2 Compression and update times

**Fig. 4.**
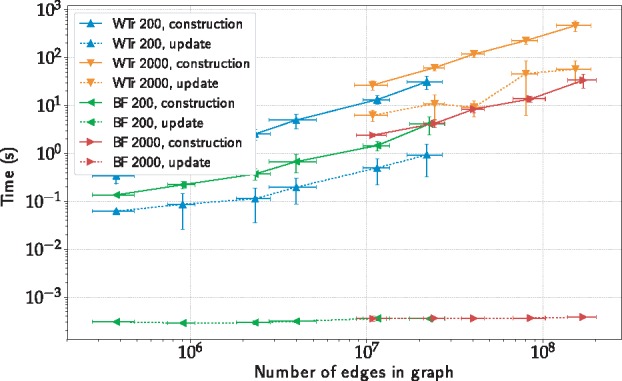
Construction vs. update times of color compressors for virus datasets of differing numbers of columns. WTr, wavelet trie; BF, Bloom filter (Color version of this figure is available at *Bioinformatics* online.)

To test the performance of updates to our dynamic compressors, we generate a set of virus datasets of increasing sizes, while keeping the numbers of columns fixed (see Supplementary Section C.1.4). Update times are an order of magnitude faster for wavelet tries and two orders of magnitude faster for Bloom filters (Fig. 4).

### 3.3 Wavelet tries and Bloom filters improve on state-of-the-art compression ratios

Finally, we close with a side-by-side comparison of the various de Bruijn graph color compression schemes presented in Section 1. In addition to these domain-specific methods, we include two popular general-purpose static compression methods, *gzip* and *bzip2*. *gzip* is an implementation of the *LZ77* algorithm that encodes blocks of text, while *bzip2* performs a sequence of transformations, including run-length encoding, BWT, move-to-front transforms, and Huffman coding.

Table 2 lists the number of bits required per edge to compress our experimental collections.

#### 3.3.1 Wavelet trie compression ratios match state-of-the-art

Our results show that wavelet trie compression outperforms *gzip* and the *VARI* method on most datasets, while performing marginally better than *Rainbowfish* and marginally worse than *bzip2* (Table 2). The *Virus100*, *Virus1000*, *Virus50000*, and *Lactobacillus* datasets are compressed to 2.2, 18.2, 662.1, and 3.3 bits per edge, respectively. The Virus1000 and Virus50000 datasets are notable in that wavelet tries without added indicator bits exhibit the worst compression ratio among the tested methods. Adding class indicator bits leads to a three-fold improvement in the compression ratio on the Virus1000 dataset (from 18.2 bits per edge to 5.3), ten-fold improvement on the *Virus50000* dataset (from 662.1 to 64.2 bits per edge), and marginal improvements in ratio on the other datasets (1.3 and 1.6 bits per edge on the *Virus100* and *Lactobacillus* datasets, respectively). In this setting, the *chr22+gnomAD* and *hg19+gnomAD* datasets are compressed to 1.2 and 5.5 bits per edge.

#### 3.3.2 Bloom filters dramatically improve on state-of-the-art

At an accuracy of 95%, our method is considerably more space efficient, achieving compression ratios over an order of magnitude greater than *bzip2* and *Rainbowfish* (Table 2). An average of 0.35 and 0.49 bits per edge are required to compress the *Virus100* and *Virus1000* datasets, respectively, compared to 5.8 and 9.7 bits for *Rainbowfish* and 4.8 and 7.5 bits for *bzip2*. An average of 2.4 bits per edge are required to compress the *Virus50000* data set, compared to 37.7 bits for *bzip2*. We were unable to compress this dataset using the *Rainbowfish* method due to its RAM consumption exceeding the per-job limit on our computing system. On the *Lactobacillus* dataset, an average of 1 bit per edge are required, compared to 7.8 bits for *Rainbowfish* and 5.7 bits for *bzip2*. On the *chr22+gnomAD* and *hg19+gnomAD* datasets, 0.45 and 0.68 bits are required per edge, compared to 2.7 and 5.4 bits for *bzip2*, and 3.3 and 5.6 bits for *Rainbowfish*.

At 99% accuracy, an increasing number of bits are required per edge with increased virus dataset size (Table 2). Fold-increases in the number of bits per edge from 1.3 bits (Virus100) to 5.4 bits (*chr22+gnomAD*) are required.

## 4 Discussion

In this study, we have addressed the problem of encoding metadata as edge colors of a given graph and demonstrated its application to de Bruijn graphs by presenting two distinct compression schemes. First, we have developed a novel application and extended parallel construction method of the wavelet trie data structure on general sequences of bit vectors that employs an iterative merging scheme to build larger tries from many smaller instances. Further, we have presented a probabilistic, compressed representation using approximate set representations that can store an arbitrary amount of annotations on the graph and allows for greater compression ratios by taking advantage of information shared between neighboring nodes to correct errors. The methods we have presented provide an important alternative to naïve static data structures for compressing binary matrices, available in libraries such as *SDSL* (Gog *et al*., 2014). Our methods allow for the dynamic addition of data and for modular combination of different colors.

We have shown that utilizing the topology of the underlying graph helps in achieving improved compression ratios. For the wavelet tries, we used indicators for the backbone regions of the de Bruijn graph positioned in prefix columns of the annotation matrix and for the Bloom filter approach, we used neighboring linear regions in the graph for error correction.

Either representation can be efficiently decompressed and queried to retrieve the coloring of arbitrary paths in the graph. Although it is helpful to know the frequency of individual colors upfront to design an optimal order of columns for the wavelet trie compression or to optimally choose the size of the individual Bloom filters used, these parameters can be easily estimated from a subsample of the input data, allowing to directly build the full coloring.

We have shown the utility of our approaches on different biological datasets, including data from virus, bacteria and human genomes, representing different graph topologies and colorings. On all datasets, we achieve comparable or strongly increased compression performance at very high levels of decompression accuracy. Notably, our approach is dynamic and allows for an easy extension with additional colors or for changes in the underlying graph structures, enabling the augmentation of large colored graphs with new annotations—a scenario commonly occurring in the genomics setting. Additionally, the wavelet trie model is fully dynamic, allowing for color and edge removal.

A possible limitation of the wavelet trie method is its reliance on shared contiguous subvectors, especially in the first few columns of the annotation matrix, to effectively partition the rows for optimal compression. The results on the viral datasets confirm that, given an annotation matrix with very sparse and mutually-exclusive rows, wavelet tries underperform relative to other methods due to tree imbalance. While this is partially addressed by setting class indicator bits in the annotation matrix, a more principled approach with less user input will become necessary in future work and could involve an analysis of the de Bruijn graph topology to algorithmically determine optimal backbone paths. Further improvements in compression ratio could be gained by an optimal ordering of the rows of the annotation matrix, but at the additional cost of maintaining a map from graph coordinates to their respective annotation matrix rows.

One of the limitations of our Bloom filter correction method is its reliance on the presence of long, identically-colored paths for correction. While this assumption worked well for the *Virus100* and *Virus1000* datasets, the shorter linear paths in the larger sets reduced our ability to correct errors in this fashion. Despite its higher compression ratio, one restriction of the Bloom filter-based method is that its corresponding graph must be accessible for reference. Although this is already done in our application, it couples color query times to graph query times. To decouple the graph from the filters, an additional structure could be constructed to indicate edges in the graph at which changes in coloring occur. Such a structure would then allow for the assumption that colors remain constant in linear regions to be relaxed.

Future work on probabilistic compression will focus on improving scaling properties. In a dynamic setting, if a dataset grows rapidly in the number of edges, the decoding accuracy will eventually drop, ultimately requiring a re-initialization into a larger Bloom filter. Further, despite being dynamic, the current probabilistic representation does not allow for the removal edges from the graph. To support this, we could replace the Bloom filters with other probabilistic set representations that allow for item removal (Bender *et al*., 2012; Fan *et al*., 2014). Lastly, an additional space improvement could be achieved with more space-efficient probabilistic set representations such as compressed Bloom filters (Mitzenmacher, 2001).

## Supplementary Material

Supplementary Material
